# Bridging Gaps in Pain Management: The Effectiveness of Educational Intervention for Nurses in a Teaching Hospital of Low- and Middle-Income Countries

**DOI:** 10.1155/nrp/8874509

**Published:** 2025-02-12

**Authors:** Ali Sarfraz Siddiqui, Tabassum Zehra, Gauhar Afshan, Sara Shakil, Aliya Ahmed

**Affiliations:** ^1^Department of Anaesthesiology, The Aga Khan University, P.O. Box 3500, Stadium Road, Karachi 74800, Pakistan; ^2^Department of Education Development, The Aga Khan University, P.O. Box 3500, Stadium Road, Karachi 74800, Pakistan

**Keywords:** epidural analgesia, hybrid educational course, initial treatment, nursing staff, pain assessment, patient-controlled analgesia

## Abstract

**Background:** Regular on-the-job training and educational courses may improve nurses' knowledge and practice regarding pain assessment and initial treatment.

**Objectives:** To assess the impact of a newly developed educational course in terms of improvement in knowledge regarding pain assessment and initial treatment by using pretest and posttest multiple-choice question scores and to determine the retention of knowledge 3 months after the educational course and perceived change in clinical practices among nurses working in adult surgical and medical wards at the Aga Khan University Hospital.

**Methods:** After getting approval from the institutional review committee, an education course was developed and conducted. A total of 86 participants attended both online and physical components. Teaching methodologies included online lectures, small group tutorials and hands-on workshops using demonstrations of locally developed videos. Pretests and posttests were conducted to assess the knowledge. All participants were requested to complete an online questionnaire to know the impact of the course on their clinical practice.

**Results:** Eighty-six participants completed the course, of which 52 (60.5%) were female and 34 (39.5%) were male. The mean difference between the pretest and posttest scores of participants was 4.72 (39.66%), which was statistically significant (*p* ≤ 0.001). The mean posttest multiple-choice question score of participants was 16.70 ± 3.56, and the mean posttest multiple-choice question score of participants after 3 months was 15.60 ± 4.09 with a mean difference of 1.10 (6.59%), which was not statistically significant (*p*=0.121).

**Conclusion:** The hybrid educational course had a significant learning impact on the knowledge of participants and their clinical practice.

## 1. Introduction

Nurses are closely involved with patients and are often the first to respond to patient issues. Ward nurses play a crucial role in the regular assessment and timely treatment of pain, as well as in managing the side effects of analgesics. Therefore, they need to have adequate knowledge and awareness regarding postoperative pain [1]. Sayed et al. found that most nursing staff (93.8%) have poor awareness of pain assessment and its treatment. They proposed that on-the-job training and regular refresher courses are necessary for nursing staff to enhance their knowledge, clinical skills and overall patient care [2].

Acute pain is a prevalent issue with various causes including surgeries, trauma, childbirth, burns, medical conditions and natural disasters. In low- and middle-income countries (LMICs), healthcare staff often lack education on the importance of pain treatment and underutilize pain medications even when available [3].

Literature showed that 75% of patients experienced moderate to severe pain in the recovery room postoperatively [4]. Data from our population indicate that the frequency of moderate-to-severe postoperative pain ranges from 51.4% to 66.8% [5]. Additionally, the prevalence of moderate-to-severe cancer pain is 62% [6] and 21.7% of patients experience persistent pain following breast cancer surgeries [7].

Inadequate postoperative pain management carries financial, physiological, psychological and ethical consequences [8]. Regular in-service training and educational courses have been shown to enhance nurse's knowledge and clinical practice. The literature also highlights the online education system's positive impact on nursing students' learning ability [9].

The primary objective of the study was to evaluate the educational course's impact on enhancing nurses' knowledge of pain management. The secondary objectives were to determine the course's impact on knowledge retention and perceived change in clinical practices 3 months after the course.

## 2. Materials and Methods

The authors designed an educational course of 5 hours of contact time on pain assessment and initial treatment. This course was delivered on five different occasions at the Aga Khan University Hospital (AKUH), a tertiary care teaching hospital in the Sindh Province of Pakistan. Approval for this experimental design: A pre- and post-(single-arm) study was granted by the Ethics Review Committee of the Aga Khan University (AKU) (ERC# 2022-7077-20401). Permission was obtained from chairs, and nursing managers, of the respective departments. Written and informed consent was obtained from all participants (nurses). Participant's name and identities were not disclosed at any time, and all information was kept confidential.

Both male and female clinical nursing instructors, registered nursing staff, nurse technician nurse interns and healthcare assistants working in the adult medical and surgical wards involved in pain management were included in this study by nonprobability purposive sampling. Participants who did not consent to participation, midwives working in the labour room, nurses working in outpatient units and nurses working in the emergency department were excluded from this study.

The theoretical framework guiding this project was Gagne's Conditions of Learning Theory [10]. To enhance the learning of participants and teaching effectiveness, course members integrated Gagne's 9 events of instruction into the teaching and learning strategies of this course [11] and social learning theory for its implementation, applying the importance of observing and imitating the behaviours (practices), attitudes, attention and retention (knowledge) and reproduction (after 3 months) [12]. The educational course had two components:

1. Online (web-based on a virtual learning environment [VLE])
2. Physical, face-to-face (hands-on workshop) (Supporting Information 1).

The duration of the online component on VLE was 90 min and that of the physical (face-to-face), hands-on workshop was 5 hours. The educational course was designed by a group of faculty members comprising four anaesthesia faculty members responsible for acute/chronic pain services, one registered pain nurse and two faculty members from the Department of Education, AKU. All course group members have been involved in lecturing and conducting educational sessions for undergraduate and postgraduate medical and nursing students. The course group considered the available evidence from the published literature in defining the content of the educational course. The online component (web-based on VLE) consists of 25 pretest MCQs as per the table of specifications, short (10 to 15 min) prerecorded presentations by subject experts and two updated articles on pain management guidelines.

Teaching methodologies included online lectures (asynchronous), small group tutorials, problem-based interactive discussion sessions using case-based clinical scenarios and hands-on workshops using demonstrations with locally developed videos and simulated patients. A uniform assessment method, comprising 25 MCQs, was used (Supporting Information 3). An online survey questionnaire was also developed to assess the educational impact of the course and the perceived change in the clinical practices of nurses 3 months after the course. The questions of the survey form were developed after the review of relevant literature and feedback from content and subject experts [13]. The questionnaire was piloted with six nurses, not included in this study before finalizing its items and content.

Written and informed consent was obtained from all participants (nurses). The courses were facilitated by faculty and staff members of the Departments of Anaesthesiology, and skills assessments were performed by the pain consultants. One staff member, trained by the IT department, facilitated the online component/session, uploaded educational materials and handled troubleshooting. Before starting the workshop, all facilitators and assessors were briefed about the assessment process and clinical skills assessment tools used. All registered participants were divided into five groups of approximately 20 members each comprising a mix of junior and senior participants. The IT Department staff of the AKU uploaded all learning materials to the VLE and monitored the activity throughout the course. After enrolling on VLE via their login ID, participants were asked to attempt pretest MCQs first with a 30-min time restriction. Participants who completed the pretest MCQs were given access to read articles and listen to prerecorded lectures.

In total, five hybrid educational courses were conducted over three months (March to May 2022) involving 86 nursing staff members from adult medical and surgical units of the AKUH. These workshops were conducted at the AKU with the support of pain medicine faculty members and pain nursing staff from the Department of Anesthesiology, AKU. All participants who completed both components (online and hands-on workshop) of the course were contacted 3 months after the course via email. They were requested to take online MCQ tests (the same MCQs were used in both pre- and posttest but with different sequences of MCQ for each individual and the test was time-bound) to assess their knowledge retention after 3 months. All participants were asked to complete an online questionnaire (Supporting Information 2) to evaluate the impact of the educational course and perceived change in their clinical practice of pain management.

### 2.1. Statistical Analysis

Data from precourse and postcourse assessments were entered into SPSS, Version 19.0 (Inc., Chicago, IL, USA), and classified under different domains for pain assessment and initial treatment. Only participants who completed both course components and both pre- and posttest MCQs were included in the analysis; the rest were excluded as missing data. The pre- and postcourse composite scores were calculated by summing the scores for each domain's questions.

Mean and standard deviation were estimated for descriptive analysis for each domain as well as overall performance. The mean differences were then computed along with their 95% confidence intervals (CIs). The Shapiro–Wilk and Kolmogorov–Smirnov tests were used to ensure that the distribution of all variables was normal. Based on the asymmetric distribution, the paired Wilcoxon signed-rank test was used to compare and determine whether there was a statistically significant difference in the pretest and posttest scores.

A *p* value of 0.05 was considered the significant threshold. The data were analysed using RStudio 4.1.2 (R Foundation for Statistical Computing).

## 3. Results

The reliability or internal consistency of MCQs used in this course was strong, with a Cronbach's alpha of 0.79 and 95% CI of 0.70 to 0.84, indicating approximately 80% reliability. A total of one hundred and six (106) nursing staff were enrolled in the educational course and ninety-two (92) attended the online component of the courses. Eighty-six (*n* = 86, 93.4%) out of 92 completed hands-on workshops. Both pretest and posttest MCQs were completed by 86 participants, of which 52 (60.5%) were female and 34 (39.5%) were male. The nursing staff included registered nurses, assistant head nurses, clinical nurse instructors, nurse technicians and healthcare assistants who worked closely with patients in adult medical and surgical wards who attended the course (Table 1).

**Figure 1:**
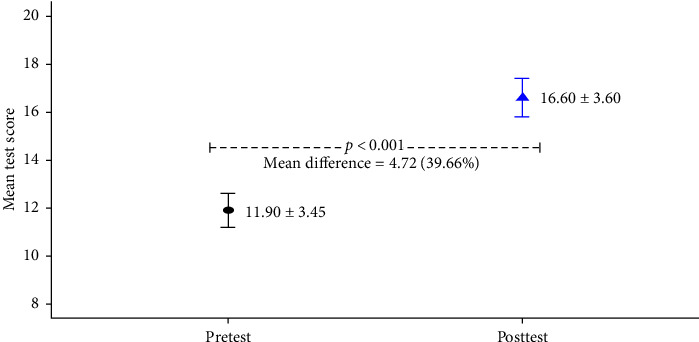
Comparison of pre- and posttest mean MCQ scores of participants (*n* = 86).

Participants' mean pretest and posttest MCQ scores were statistically significant (*p* ≤ 0.001). (Figure 1).

Individual pre- and posttest scores were not compared. However, a comparison of the mean pre- and posttest MCQ scores of participants according to specific topic/session showed statistically significant improvement (*p* ≤ 0.001) (Table 2).

The impact of the educational course was assessed 3 months after conducting the physical component (workshop) by administering the posttest MCQs and online survey questionnaires. A total of 74 nursing staff completed the second posttest MCQs assessment and online questionnaire. The mean MCQs test score of 74 participants immediately after the workshop was 16.70 ± 3.56. After 3 months, the mean posttest MCQs score was 15.60 ± 4.09, resulting in a mean difference of 1.10 (6.59%); 95% CI for this difference was 2.37–0.12, which was statistically nonsignificant (*p*=0.121). The overall decline in knowledge three months after the educational course was 6.59%. Comparison of MCQ scores immediately posttest and after three months showed that mean MCQs scores for specific topic/session were not statistically significant, except for persistent postsurgical pain (*p*=0.001) and clinical decision-making and troubleshooting (*p* ≤ 0.001).

In the univariate analysis, the effect of educational courses was significantly higher in the male gender (*p*=0.041) and among participants less than 30 years of age (*p*=0.008). Participants from the postanaesthesia recovery unit showed less improvement in knowledge compared to the surgical and medical ward nurses, but this difference was not statistically significant (*p*=0.373).

Nurse technicians and healthcare assistants showed less improvement in knowledge compared to the registered nurses, assistant head nurses and clinical nurse instructors, though this difference was also not statistically significant (*p*=0.543). In the multivariate analysis, the linear regression coefficient for participants under 30 years of age showed more improvement in posttest scores compared to those over 30 years of age, but this was not statistically significant (*p*=0.060) (Table 3)

In the univariate analysis, the decline in knowledge three months after the educational course was significantly higher in the male gender (*p*=0.001) and in those participants who worked in postanaesthesia recovery unit (*p* ≤ 0.001). Nurse technicians and healthcare assistants showed more decline in knowledge as compared to the registered nurses, assistant head nurses and clinical nurse instructors, but this difference was not statistically significant (*p*=0.184).

In the multivariate analysis, the linear regression coefficient for participants who were male showed more decline in posttest scores after three months as compared to females (*p*=0.014) and those who worked in postanaesthesia recovery unit showed more decline in posttest scores after three months as compared to the surgical and medical ward nurses with statistically significant values (*p*=0.005) (Table 4)

Results from the online survey questionnaire showed that sixty-four (*n* = 64) 86.5% of participants strongly agreed or agreed that their knowledge of pain assessment has improved after the educational course. Regarding the utilization of knowledge acquired during the course in their clinical practice, participants responded that they would use knowledge in pain assessment, initial treatment of pain, patient care, documentation, clinical decision-making, use of multimodal analgesia, early reporting to primary team members, proper use of pain scale and timely intervention to manage patient using PCIA or epidural infusion. In response to the question regarding the components of the course that had the most impact on improving their knowledge, the participant mentioned the online module, video-based interactive discussion and clinical assessment of patients/hands-on practice sessions as the most useful.

Sixty-five (*n* = 65, 87.8%) participants strongly agreed or agreed that their clinical skills in pain assessment have improved after the educational course. Sixty-six (*n* = 66, 89.18%) participants strongly agreed or agreed that their clinical skills of initial pain treatment have improved after the educational course. Sixty-five (*n* = 65, 87.83%) participants strongly agreed or agreed that this course was useful in the clinical management of patient with PCIA. Sixty-one (*n* = 61, 82.43%) participants strongly agreed or agreed that this course was useful in the clinical management of patient with epidural infusion.

In response to a question regarding whether pain assessment improves pain management and patient satisfaction, participants responded that pain is a subjective experience of the individual patient. Recognizing and assessing pain using appropriate pain scale is essential for planning pain management modalities. Timely identification can lead to early initiation of pain treatment. Appropriate pain management helps in early recovery and early mobilization and prevents complications, which in turn increases patient and family satisfaction. In response to a question about recommending this educational course to other nursing staff, sixty-four (*n* = 64, 86.48%) participants strongly agreed or agreed that they would recommend this educational course to their colleagues.

Overall, the participants provided very positive and encouraging remarks about this educational activity. Many suggested that such activity should be conducted regularly for all nursing staff and recommended that this training be included in the nursing education curriculum and the induction process for all nursing staff.

## 4. Discussion

To the best of the authors' knowledge, this was the first hybrid education course on pain assessment and initial treatment developed and conducted for nursing staff working in adult surgical and medical wards in Pakistan. The effectiveness of the course was demonstrated by the statistically significant increase in knowledge of the participants after attending the course (*p* ≤ 0.001). This knowledge improvement and enhanced proficiency will help nurses to apply their knowledge in better patient care leading to improved patient outcomes and increased patient satisfaction. Acute postoperative pain management remains a significant challenge for physicians and nursing staff globally despite several studies, better pain management education programs, availability of new methods, strong analgesics and clinical practice guidelines [14].

Monakshi et al. examined the feasibility and effectiveness of simulation-based education for registered nurses in managing patients receiving epidural analgesia. They showed a significant improvement in epidural knowledge after the workshop, an increase in the correct procedures performed during observation and enhanced skill in assessing sensory and motor blocks. The nurses were also better at relating the clinical findings to their assessment and recognizing the adverse effects of epidural analgesia [15].

Kasahun et al. reported that 70% of adult patients experience moderate to severe pain following elective surgery [16]. Effective management is crucial as inadequate pain relief may lead to significant morbidity [17]. Sharma et al. recommend routine pain assessment and documentation alongside other vital signs to enhance postoperative pain management [18].

In this study, the hybrid educational course demonstrated effectiveness with a significant 39.66% improvement in nursing staff knowledge after attending the course. The participant's mean pretest MCQ score was 11.90 ± 3.45, which increased to 16.60 ± 3.60, post-test, which was statistically significant (*p* ≤ 0.001). In a similar study by Uysal et al. [19], the average knowledge score of nurses before and after the educational session was 0.67 ± 0.15 and 0.81 ± 0.13, respectively, which was statistically significant, and the nurse clinical practices for pain management before and after the educational session were also statistically significant in some respects (*p* ≤ 0.005).

Recent literature showed that after the implementation of the pain assessment and management program for nurses, statistically significant improvements were noted in the knowledge of nurses, and their responses after the intervention were improved significantly [20–22]. Studies demonstrated that following the implementation of a web-based educational program for nurses, the clinical knowledge of nursing staff and the quality of postoperative pain management of patients showed a significant improvement [23, 24].

Lucia et al. evaluated knowledge of pain assessment and treatment in nurses trained via the online module (video-based) using MCQs. They showed that nurse's knowledge improved after the training session with significant improvement in clinical skills [25]. In this study, the online component of the course was developed on VLE containing video-recorded lectures and updated articles for reading and during the course, a video-based teaching method was used to educate nurses about bedside clinical skills.

Similar studies using in-service education programs consisting of several educational methods like video-based learning and case-based discussion and providing teaching and learning materials showed a significant improvement in nurses' knowledge and attitudes about pain management after attending the program [26, 27].

Parvizy et al. mentioned that nurses are influenced by the attitude of patients in managing pain. They observed a significant improvement in their knowledge and attitude after 4 hours of educational sessions. They showed that 81% of nurses believed that patients overexpress their pain and incorrect judgement of ward nurses indicated their negative attitude towards pain assessment [28].

An interval assessment was carried out 3 months after the educational course to evaluate the retention of knowledge, which showed a slight decline of 6.59% in knowledge 3 months after the educational course, indicating the ongoing positive impact of the educational course. Aminoroaia et al. showed a significant increase in nurses' knowledge, and clinical care, immediately after and 3 months after the educational intervention [29]. Chun-Hua Zhang et al. and others showed improvement in nurses' knowledge and attitudes towards pain management of patients after the implementation of the Pain Education Program (PEP). The ability to use Changhai Pain Scale to assess patients' pain intensity was significantly improved after the PEP [30, 31].

Sixty-four (86.5%) participants strongly agreed or agreed that their knowledge of pain assessment has improved after the educational course. Sixty-five (87.8%) participants strongly agreed or agreed that their clinical skills in pain assessment have improved after the educational course. Sixty-five (87.83%) participants strongly agreed or agreed that this course was useful in the clinical management of patient with PCIA. Sixty-one (82.43%) participants strongly agreed or agreed that this course was useful in the clinical management of patient with epidural infusion. Lucia et al. conducted an e-learning course for nurses on pain assessment and showed that nurses were highly satisfied with the training session, the course media, methodology and learning material, the knowledge acquired and the ability to apply knowledge to routine clinical practice [25].

## 5. Conclusion

The hybrid educational course showed statistically significant improvement and impact on the participants' knowledge after attending the course. The results indicated a significant improvement in the posttest scores of the majority of participants. There was only a slight decline in participants' overall knowledge, 3 months after the educational course.

## 6. Strengths

To the best of the researcher's knowledge, this was the inaugural course developed and conducted in Pakistan. This course not only assesses the baseline knowledge but also assessed the change in knowledge of participants after the educational course. In this study, retention of knowledge and perceived change in clinical practices among nurses working in adult surgical and medical wards were also observed, 3 months postintervention.

## 7. Limitations

The absence of control group (without intervention) may limit the ability to conclude the observed intervention effect. Therefore, conducting hybrid educational courses and workshop-based training will hopefully be the beginning of a practice change in pain assessment that may lead to appropriate pain treatment for admitted patients. It is important to note that this study is limited by a single-centre design involving nurses from a single institution; thus, caution is needed in generalizing the results.

## 8. Recommendations

The nursing staff responsible for managing hospitalized patients should undergo regular training sessions using standardized pain assessment tools. Regular pain assessment is recommended for optimal care and a better outcome for patients. Such educational courses should be regularly conducted for better understanding and implementation in hospital settings. It is recommended that these educational courses be conducted in all tertiary care and teaching hospitals; integrating this educational course into the orientation sessions for all nursing staff could further enhance its effectiveness.

## 9. Implications

Significant improvement in participants' knowledge after attending the course is a promising indicator for improvement in the pain management of hospitalized patients. Effective utilization of this knowledge by the participants within their respective units could position them as a peer capable of supporting and guiding other nursing staff of the hospital.

## Figures and Tables

**Table 1:** Clinical work areas, designations and years of experience of the participants (*n* = 86).

Variable	*n* (%)
*Clinical work area*	
Medical and allied ward	15 (17.4)
Postanaesthesia care unit (PACU)	31 (36.0)
Surgical and allied ward	40 (46.5)

*Designation*	
Assistant head nurse/nurse instructor	6 (7.0)
Healthcare assistant/nurse technician	17 (19.8)
Registered nurse	63 (73.3)

*Work experience (years)*	
Less than one	5 (5.8)
One to five	46 (53.5)
Five to ten	27 (31.4)
More than ten	8 (9.3)

**Table 2:** Comparison of pre- and posttest mean MCQ scores of participants according to topic (*n* = 86).

Educational session (topics)	MCQs Pretest (*n* = 86)	MCQs Posttest (*n* = 86)	Comparison of means		
Domain	Mean ± SD	Mean ± SD	Mean difference^†^	95% CI	*p* ^∗^
Anatomy and physiology of pain pathways	1.51 ± 0.89	2.10 ± 1.06	0.59 (39.07%)	0.29–0.90	< 0.001
Clinical implications and morbidities of acute pain	0.33 ± 0.52	0.77 ± 0.71	0.44 (133.33%)	0.25–0.64	< 0.001
Pain assessment modalities in conscious adult patients	2.37 ± 0.77	2.77 ± 0.45	0.40 (16.88%)	0.22–0.57	< 0.001
Persistent postsurgical pain	1.27 ± 0.85	2.41 ± 0.83	1.14 (89.76%)	0.88–1.40	< 0.001
Initial treatment of pain and multimodal analgesia	4.36 ± 1.58	5.43 ± 1.57	1.07 (24.54%)	0.65–1.49	< 0.001
Clinical decision-making and troubleshooting	2.03 ± 1.12	3.23 ± 0.90	1.20 (59.11%)	0.91–1.49	< 0.001
Overall	11.90 ± 3.45	16.60 ± 3.60	4.72 (39.66%)	3.74–5.71	< 0.001

Abbreviation: CI, confidence interval.

^†^Mean difference is presented as absolute and (%) difference.

⁣^∗^Values for pre- and posttest scores are presented as mean ± SD.

⁣^∗^*p* values were calculated by Wilcoxon signed-rank test, except overall (paired *t*-test).

**Table 3:** Univariate and multivariate analyses showing the effect of different factors on pre- and posttest scores.

Variable	Univariate models			Multivariate models		
	β (SE)	*p* -value	95% CI	Adjusted β (SE)	*p* -value	95% CI
*Age*						
< 30 years	2.98 (1.09)			2.30 (1.20)		
≥ 30 years	Ref	0.008	0.80 to 5.16	Ref	0.060	−0.10 to 4.70

*Gender*						
Female	Ref			Ref		
Male	2.28 (1.10)	0.041	−4.47 to −0.09	−1.09 (1.14)	0.339	3.36 to 1.17

*Designation*						
AHN/CNI/HN	Ref			Ref		
RN	2.98 (1.77)	0.096	−0.55 to 6.52	2.02 (1.81)	0.267	−1.58 to 5.63
NT/HCA	−1.26 (2.07)	0.543	−5.39 to 2.86	−1.87 (2.09)	0.374	−6.05 to 2.30

*Clinical area/ward*						
Surgical	Ref			Ref		
Medical	1.31 (1.83)	0.474	−2.34 to 4.97	−0.47 (1.76)	0.789	−3.99 to 3.04
PACU	−1.03 (1.16)	0.373	−3.35 to 1.27	0.12 (1.12)	0.915	−2.12 to 2.36

*Note:* Outcome = post − pretest change. Independent: age, gender, designation and clinical working area. General linear model.

Abbreviations: AHN/CNI/HN, assistant head nurse/clinical nurse instructor/head nurse; NT/HCA, nurse technician/healthcare assistant; PACU, Postanaesthesia Care Unit; RN, registered nurse.

**Table 4:** Univariate and multivariate analyses showing the effect of different factors on scores of posttests and posttests after 3 months.

Variable	Univariate models			Multivariate models		
	β (SE)	*p* value	95% CI	Adjusted β (SE)	*p* value	95% CI
*Age*						
< 30 years	0.95 (1.07)			−0.64 (1.07)		
≥ 30 years	Ref	0.377	−1.18 to 3.07	Ref	0.553	−2.78 to 1.50

*Gender*						
Female	Ref			Ref		
Male	−3.25 (0.98)	0.001	−5.21 to −1.29	−2.55 (1.01)	0.014	−4.58 to −0.53

*Designation*						
AHN/CNI/HN	Ref			Ref		
RN	0.62 (1.71)	0.716	−2.78 to 4.02	0.61 (1.61)	0.706	−2.61 to 3.83
NT/HCA	−2.67 (1.99)	0.184	−6.64 to 1.30	−1.65 (1.87)	0.380	−5.37 to 2.08

*Clinical area/ward*						
Surgical	Ref			Ref		
Medical	−0.71 (1.58)	0.656	3.87 to 2.45	−1.32 (1.57)	0.404	−4.46 to 1.82
PACU	−3.67 (1.00)	< 0.001	−5.66 to −1.67	−2.92 (1.00)	0.005	−4.92 to −0.92

*Note:* Outcome = post − -3-month change. Independent: age, gender, designation and clinical working area. General linear model.

Abbreviations: AHN/CNI/HN, assistant head nurse/clinical nurse instructor/head nurse; NT/HCA, nurse technician/healthcare assistant; PACU, Postanaesthesia Care Unit; RN, registered nurse.

## Data Availability

The data supporting this study's findings are available from the corresponding author upon reasonable request.

## Supporting Information

Supporting Information 1 Workshop program: Five workshops were conducted for the participants according to this program with the support of pain medicine faculty members and pain nursing staff from the Department of Anesthesiology, AKU.

Supporting Information 2 Online questionnaire: An online survey questionnaire was used to assess the educational impact of the course and the perceived change in the clinical practices of nurses 3 months after the course.

Supporting Information 3 Assessment tool (MCQs).

Supporting Information 4 SQUIRE checklist: The manuscript has been prepared according to the guidelines in the SQUIRE checklist for reporting quality improvement in healthcare [32].
