# Supplementary material for: Bridging Gaps in Pain Management: The Effectiveness of Educational Intervention for Nurses in a Teaching Hospital of Low- and Middle-Income Countries
Source: Nurs Res Pract. 2025 Feb 12;2025:8874509. doi: 10.1155/nrp/8874509 (PMC11839259; doi:10.1155/nrp/8874509)
Supplement: Supporting Information 1 — Workshop program: Five workshops were conducted for the participants according to this program with the support of pain medicine faculty members and pain nursing staff from the Department of Anesthesiology, AKU. [file 8874509.f1.pdf]

Workshop Program

SNo.	Topic	Teaching Modalities
1.	Introduction to the course and participants Brainstorming session	Interactive session
2.	Pain assessment of adult patients with Epidural / PCIA	Clinical skills assessment using Likert scale
3.	Pain assessment and assessment of a patient with Epidural / PCIA	Video-based Interactive session and hands-on session
4.	Troubleshooting of patients with Epidural and PCIA	Case-based learning
5.	Pain assessment of adult patients with Epidural / PCIA	Sign off with the Likert scale
6.	Debriefing session	
7.	Post-test	MCQs
8.	Feedback and course evaluation	
