# Supplementary material for: Bridging Gaps in Pain Management: The Effectiveness of Educational Intervention for Nurses in a Teaching Hospital of Low- and Middle-Income Countries
Source: Nurs Res Pract. 2025 Feb 12;2025:8874509. doi: 10.1155/nrp/8874509 (PMC11839259; doi:10.1155/nrp/8874509)
Supplement: Supporting Information 2 — Online questionnaire: An online survey questionnaire was used to assess the educational impact of the course and the perceived change in the clinical practices of nurses 3 months after the course. [file 8874509.f2.pdf]

Pain assessment and initial treatment: Effectiveness of a hybrid educational course for nursing staff in a tertiary care hospital

Questionnaire for three months survey post course

Serial # ………..

Demographics:

1. Age:………….years 2. Gender: female / male

3. Designation: ………………………….

4. Year of clinical experience: ……… years

5. Clinical working area: ……………………………..

Educational impact of the course:

To what extent do you agree with the following statement,

1. Your knowledge about pain assessment has improved after the educational course

   Strongly Disagree Neutral Agree Strongly Agree

2. How do you utilize acquired knowledge during the course into your clinical practice?

   ……………………………………………………………………………………

   ……………………………………………………………………………………

3. Which component of this course in your opinion had most impact in improving your clinical knowledge?

   ………………………………………………………………………………….

Perceived clinical impact of the course:

To what extent do you agree with the following statement,

1. Your clinical skills of pain assessment have improved after the educational course

   Strongly Disagree Neutral Agree Strongly Agree

2. Your clinical skills of initial pain treatment have improved after the educational course

   Strongly Disagree Neutral Agree Strongly Agree

3. You find this course useful for you in the clinical management of patient with PCIA

   Strongly Disagree Neutral Agree Strongly Agree

4. You find this course useful for you in the clinical management of patient with continuous infusion via Epidural catheter

   Strongly Disagree Neutral Agree Strongly Agree

5. In your opinion, how does pain assessment improve pain management and patient satisfaction?

   …………………………………………………………………………………………

6. You will recommend this educational course to other nursing staff

   Strongly Disagree Neutral Agree Strongly Agree

7. Which component of this course in your opinion had the most impact on improving your clinical skills?

   …………………………………………………………………………………………

8. Did you face any challenge while taking this educational course?

   Yes No

   If yes, please mention

   …………………………………………………………………………………………

   ……………………………………………………………………………………..

Any comment / suggestion:

…………………………………………………………………………………………

…………………………………………………………………………………………
