# Supplementary material for: Bridging Gaps in Pain Management: The Effectiveness of Educational Intervention for Nurses in a Teaching Hospital of Low- and Middle-Income Countries
Source: Nurs Res Pract. 2025 Feb 12;2025:8874509. doi: 10.1155/nrp/8874509 (PMC11839259; doi:10.1155/nrp/8874509)
Supplement: Supporting Information 3 — Assessment tool (MCQs). [file 8874509.f3.pdf]

Pain assessment and initial treatment: A basic certificate course for nursing staff

Name of Participant: __________________ Department /Area of work: __________

Designation: _____________________________ Date _________________

MCQs Paper (Single best)

1. **A 25-year-old man was admitted to the post-operative ward after right shoulder surgery. He had severe pain in his right shoulder.**

   The physiological process involved in the conduction of pain signals to the brain is:

   A. Modulation
   B. Perception
   C. Transcription
   D. Transduction
   E. Transmission

2. **A 30-year-old male patient with no known comorbid was admitted to the post-operative unit after right inguinal hernia repair.**

   For his pain assessment, the most appropriate pain scale used clinically is**:**

   A. Bromage scale
   B. Faces pain scale
   C. Numeric rating scale
   D. Behavioral pain scale
   E. Visual analogue scale

3. **A 35-year-old male patient was admitted to the post-operative ward after right inguinal hernia repair under general anaesthesia. He has moderate pain (NRS 6/10) on movement.**

   His pain signal is conducted from the site of injury to the brain by:

   A. Fast conducting myelinated C fibers
   B. High velocity myelinated Aδ fibers
   C. High-velocity myelinated B fibers
   D. Slow conducting Aδ fibers
   E. Slow conducting C fibers

4. **A 35-year-old male was admitted to the post-operative ward after repair of right inguinal hernia under general anesthesia. Post-operatively he developed moderate pain (NRS = 5/10) over the surgical site that was managed by multimodal analgesia and using a physical modality.**

   The most effective physical modality for this patient would be:

   A. Acupuncture
   B. Cold Therapy
   C. Localized heat
   D. Massage therapy
   E. TENS therapy

5. **A 40-year-old male patient with no known comorbid presented to the pain clinic with moderate pain over the right inguinal region for the last nine months. He has a history of right inguinal hernia repair two years back and right recurrent inguinal hernia repair with a mesh one year back.**

   The factor that has most likely resulted in persistent post-surgical pain in this patient is:

   A. Age of patient
   B. Gender of patient
   C. Repeat surgery
   D. Site of surgery
   E. Type of surgery

6. **A 45-year-old female was admitted to the postoperative unit after debulking gynecological surgery. Intravenous paracetamol and bupivacaine 0.1% at 12 ml/hour via epidural catheter were given for pain management. On examination, she is vitally stable and pain-free but unable to bend her right knee and able to move her ankle joint.**

   Your next step of management for the right knee motor block would be:

   A. Continue same treatment and reassess after four hours
   B. Raise her right leg to 45° and reassess after four hours
   C. Stop epidural infusion and reassess after four hours
   D. Turn the patient, keep left side up and reassess after two hours
   E. Turn the patient, keep right side up and reassess after two hours

7. **A 45-year-old female was admitted to the postoperative unit after exploratory laparotomy. Bupivacaine 0.1% at 10 ml/hour was given via epidural catheter for pain management. On examination, she is vitally stable but unable to bend her right hip and knee and only able to move her ankle joint.**

   Using modified Bromage scale, her right lower limb motor block score is:

   A. 1/3
   B. 2/3
   C. 3/3
   D. 2/10
   E. 5/10

8. **A 45-year-old male patient was admitted to the post-operative unit after exploratory laparotomy. He repeatedly complained of moderate to severe pain (NRS 6-8/10) which was managed with I/V boluses of tramadol and paracetamol.**

   The most likely reason for not using strong opioids in this patient by the healthcare team is:

   A. Careless attitude of nursing staff
   B. Chance of addiction to analgesic drugs
   C. Fear of complications with strong opioids
   D. Lack of awareness about pain management among nursing staff
   E. Poor assessment of pain intensity

9. **A 45-year-old male patient was admitted with severe pain in the abdomen. He had a history of stomach carcinoma. His pain was managed with I/V boluses of morphine and I/V paracetamol and then a continuous infusion of 2mg/hour of morphine started. After four hours patient became drowsy. On examination, the patient is vitally stable, SpO2, = 94%, RR=10/min but he is drowsy.**

   The next management step for this patient would be:

   A. Administer oxygen with face mask and continue the same treatment
   B. Hold morphine, administer oxygen, and monitor the patient
   C. Monitor patient’s vitals and continue the same treatment
   D. Stop opioids and give a single bolus of I/V naloxone
   E. Stop opioids and give repeated boluses of I/V naloxone

10. **A 45-year-old male weighing 70kg was prescribed patient-controlled analgesia (PCA) for postoperative pain management after exploratory laparotomy. The prescription included a background infusion of Morphine 1mg/hour, a bolus of 1mg, and a lockout time of 10 minutes.**

    The part of the information which will mainly determine the success of PCA is:

    A. Background infusion
    B. Bolus dose
    C. Lockout interval
    D. Type of surgery
    E. Weight and age

11. **A 50-year-old female was admitted to the postoperative unit after gynecological debulking surgery. Intravenous paracetamol and bupivacaine 0.1% at 12 ml/hour via epidural catheter were given for pain management. On examination, she is pain-free, but her BP is 85/55 mmHg, HR is 95 b/m, and SpO_2, = 94%.**

    Your next step in the management would be:

    A. Give intravenous fluid bolus of 500 ml and record vitals every 15 minutes
    B. Give intravenous fluid bolus of 500 ml, vasopressor, and call duty physician
    C. Give oxygen, intravenous fluid bolus of 500 ml and vasopressor
    D. Stop epidural infusion, give O_2, 200 ml fluid bolus and inform physician
    E. Stop epidural infusion, give vasopressor and inform duty physician

12. **A 50-year-old male patient was admitted to the post-operative unit after a laparotomy. He is getting I/V paracetamol and tramadol infusion at 10 mg/hour. On assessment, he has moderate pain (NRS 6/10) in the abdomen.**

    Your next step of management would be to:

    A. Apply TENS therapy around surgical site
    B. Contact physician for rescue analgesia
    C. Continue same intravenous analgesic agents
    D. Give 5 mg bolus dose of intravenous morphine
    E. Provide patient education about pain

13. A 50-year-old male underwent an emergency below-knee amputation due to road traffic accident. He received multi-modal analgesia postoperatively. On his follow up visit he continued to have moderate pain over stump area for up to six months.

He was diagnosed to have persistent post-surgical pain that is defined as pain that persist after surgery for more than:

A. one month
B. two months
C. three months
D. one year
E. six months

14. A 53-year-old female on hemodialysis for renal failure undergoes emergency laparotomy for small bowel resection. Postoperatively, she received multi-modal analgesia for her pain management.

The analgesic drug that is contraindicated in this patient is:

A. Morphine
B. Nalbuphine
C. NSAIDs
D. Paracetamol
E. Tramadol

15. A 53-year-old female with renal failure and on hemodialysis undergoes emergency laparotomy for small bowel resection. Postoperatively, she received intravascular morphine of 2 mg every 6 hours. On the second postoperative day, she became very sedated, and her respiratory rate dropped to 8 breaths/min.

Your immediate action would be:

A. Attach monitors and arrange for immediate hemodialysis
B. Give Oxygen, attach monitors, and prepare Naloxone
C. Give Oxygen, attach monitors, and record vitals
D. Give Oxygen, attach monitors and send blood for ABG’s
E. Give Oxygen, call for help, and start CPR

16. **A 55-year-old male patient was admitted to the post-operative unit after laparotomy. His pain was managed with multimodal analgesia. He complains of moderate pain in the abdomen and difficulty in breathing.**

    The postoperative respiratory complication in this patient may lead to:

    A. Heart attack
    B. Constipation
    C. Pneumonia
    D. Asthma
    E. Pulmonary embolism

17. **A 55-year-old male patient was admitted to the post-operative unit after laparotomy. His pain was managed with multimodal analgesia. On routine assessment, the duty nurse uses a numerical rating scale (NRS) for pain assessment**

    Use of NRS involves asking the patient to:

    A. Assign a number to his pain intensity
    B. Categorize the intensity of his pain
    C. Describe characteristics of pain
    D. Inform about the duration of pain
    E. Mark a point on a straight line

18. **A 55-year-old male patient was admitted to the postoperative ward after right shoulder arthroplasty. He was given intravenous morphine patient-controlled analgesia (PCA), paracetamol, and NSAIDs for pain management. Six hours later he developed nausea followed by two episodes of vomiting and his respiratory rate slows down to 10 breaths per minute.**

    The most likely cause of the above complications is:

    A. Age of patient
    B. Intravenous NSAIDs
    C. Intravenous paracetamol
    D. Operation performed
    E. Use of morphine PCA

19. **A 55-year-old male patient weighing 60kg was prescribed PCA for postoperative pain management after laparotomy. He has prescribed a background infusion of Morphine 1mg/hour, a bolus of 1mg, and a lockout time of 8 minutes. On the second postoperative day, he was heavily sedated and difficult to be awake and his PCA demand was 8 and delivered dose was 8.**

    The most likely cause of the above complications is:

    1. Age and Weight
    2. Background infusion
    3. Bolus dose
    4. Lockout interval
    5. Type of surgery

20. **A 60-year-old female patient was admitted to the postoperative ward after right knee arthroplasty. The surgeon had infiltrated the local anaesthetic at the incision site and the patient was receiving intravenous morphine patient-controlled analgesia (PCA), paracetamol, and NSAIDs for pain management.**

    The pain management strategy employed in this patient is:

    A. Adjuvant drug therapy
    B. Co-analgesic therapy
    C. Multimodal analgesia
    D. Pre-emptive analgesia
    E. Regional analgesia

21. **A 60-year-old male patient was admitted to the post-operative unit after laparotomy. He is a known case of uncontrolled hypertension and diabetes mellitus for the last ten years. He complains of severe pain in the abdomen (NRS 8/10).**

    The most likely complication due to severe postoperative pain in this patient would be:

    A. Arrhythmia
    B. Constipation
    C. Heart attack
    D. Hypertension
    E. Pulmonary embolism

22. A 60-year-old male with chronic renal failure and uncontrolled diabetes mellitus undergo an emergency below-knee amputation. Multi-modal analgesia and bupivacaine 0.1% at 5 ml/hour were given via peri-neural catheter for postoperative pain management.

The drug that blocks the transmission of pain signals from periphery to brain in this patient is:

A. Bupivacaine
B. Nalbuphine
C. NSAIDs
D. Paracetamol
E. Tramadol

23. A 70-year-old female patient was admitted to the postoperative ward after left total knee replacement. The surgeon had infiltrated bupivacaine 0.25% with NSAIDs at the wound site and patient was receiving intravenous morphine patient-controlled analgesia (PCA), paracetamol, and NSAIDs for pain management.

The drug that had the most effect on transduction process in this patient during immediate postoperative period is:

A. Bupivacaine
B. I/V NSAIDs
C. Local NSAIDs
D. Morphine PCA
E. Paracetamol

24. In post-operative wards, five vital signs are assessed and documented to monitor the patient's condition

A fifth vital sign that is assessed is:

A. Blood pressure
B. Heart rate
C. Respiratory rate
D. Pain severity
E. Temperature

25. Multimodal perioperative pain management is often suggested as a preventive strategy to reduce the incidence of chronic pain after surgery, but its incidence is variable after different surgeries.

Operation with the expected highest incidence of persistent post-surgical pain one year after surgery is,

A. Amputation of limb
B. Inguinal hernia repair
C. Laparotomy
D. Mastectomy
E. Sternotomy
